# Effect of argon plasma pre-treatment of healing abutments on peri-implant microbiome and soft tissue integration: a proof-of-concept randomized study

**DOI:** 10.1186/s12903-023-02729-1

**Published:** 2023-01-17

**Authors:** Luigi Canullo, Mia Rakic, Emilio Corvino, Maria Burton, Janina A. Krumbeck, Aishani Chittoor Prem, Andrea Ravidà, Nenad Ignjatović, Anton Sculean, Maria Menini, Paolo Pesce

**Affiliations:** 1 Department of Periodontology, University of Bern, Bern, Switzerland; 2 Department of Surgical Sciences (DISC), University of Genoa, Genoa, Italy; 3 ETEP (Etiology and Therapy of Periodontal Diseases) Research Group, University Complutense of Madrid, Madrid, Spain; 4 University of Florence, Florence, Italy; 5 Zymo Research Corporation, 17062 Murphy Ave, Irvine, CA 92614 USA; 6 Pangea Laboratory, 14762 Bentley Cir., Tustin, CA 92780 USA; 7 Department of Periodontology, Ann Harbor, MI USA; 8 Institute of Technical Science of the Serbian Academy of Sciences and Arts, 11000 Belgrade, Serbia

**Keywords:** Biofilms, Microbiome, Dental implants, Argon plasma, Healing abutments, Soft tissues

## Abstract

**Purpose:**

Biofilm-free implant surface is ultimate prerequisite for successful soft and bone tissue integration. Objective of the study was to estimate the effects of argon plasma healing abutment pre-treatment (PT) on peri-implant soft-tissue phenotype (PiSP), inflammation, plaque accumulation and the microbiome (PiM) between non-treated (NPT) and treated (PT) abutments following 3-months healing period. The hypothesis was that cell-conductive and antimicrobial properties of PT would yield optimal conditions for soft tissue integration.

**Material and Methods:**

Two months following second-phase surgery, microbiological and clinical parameters were assessed around thirty-six healing abutments with two types of microtopography, smooth surface (MACHINED) and ultrathin threaded microsurface (ROUGH). A two level randomization schema was used to achieve equal distribution and abutments were randomly divided into rough and machined groups, and then divided into PT and NPT groups. PiM was assessed using next-generation DNA sequencing.

**Results:**

PiM bacterial composition was highly diverse already two months post-implantation, consisting of key-stone pathogens, early and late colonizers, while the mycobiome was less diverse. PT was associated with lower plaque accumulation and inflammation without significant impact on PiSP, while in NPT clinical parameters were increased and associated with periopathogens. NPT mostly harbored late colonizers, while PT exerted higher abundance of early colonizers suggesting less advanced plaque formation. Interaction analysis in PT demonstrated *S. mitis* co-occurrence with pro-healthy *Rothia dentocariosa* and co-exclusion with *Parvimonas micra*, *Porphyromonas endodontalis* and *Prevotella oris.* PiSP parameters were generally similar between the groups, but significant association between PiM and keratinized mucosa width was observed in both groups, with remarkably more expressed diversity in NPT compared to PT. PT resulted in significantly lower BOP and PI around rough and machined abutments, respectively, without specific effect on PiM and PiSP.

**Conclusions:**

PT contributed to significantly the less advanced biofilm accumulation and inflammation without specific effects on PiSP.

**Supplementary Information:**

The online version contains supplementary material available at 10.1186/s12903-023-02729-1.

## Introduction

Implant therapy is a gold standard treatment for restoration of missing teeth, based on outstanding functional and aesthetic properties it provides to the patient, while preserving adjacent teeth and improving the oral health-related quality of life [1]. However, the constant exposure of dental implants to the abundant oral microflora may affect the quality of the soft and bone tissue integration, while increasing their susceptibility to peri-implant infection. Peri-implantitis is defined as a chronic inflammatory disease of peri-implant tissues, which is induced by multiple etiologic factors, and in particular by dysbiotic bacterial biofilms accumulated on implant surface [2]. This disease is very burdensome for doctors and patients and with tremendous growth of the implant market. Consequentially, peri-implantitis is currently qualified as emerging public health problem due to increasing prevalence (12–45%) and lack of predictive treatment [3, 4]. Therefore, the prevention of peri-implant infection remains the major priority in management of implant patients [5, 6].

Since soft periodontal and peri-implant tissue are the first line defenders against infective threats (60), it is understandable that lack of periodontal ligament comprehensibly represents the *Achilles heel* of dental implants making them more susceptible to infection and related immunopathologies [8, 9]. The key-elements of the peri-implant soft tissue phenotype (PiSP) responsible for long-term implant success are mucosal thickness (MT), keratinized mucosa width (KMW), and/or supracrestal height (STH) [10]. In context of long-term peri-implant stability, thick tissues and wider KMW better preserve soft and hard peri-implant tissues, by preventing recession, bacterial accumulation and apical penetration, while also stabilizing peri-implant bone remodeling [11–13]. However, the PiSP is site-specific and susceptible to changes over the time, as well as environmental factors [10]. In this context, the microbial factors lately receive an increasing attention. Besides its precursor role in future infections, the low-grade inflammation associated with implant surface colonization negatively modulates the healing processes within implant integration affecting its respective solidity and functionality [7, 14]. In brief, the biofilm formation on implant surfaces followed by colonization by pre-existing oral bacterial species occurs within the first minutes of post-implantation. This process is additionally facilitated by implant surface micro and macro topography [15]. The pre-existing species also consist of keystone periopathogens, the inflammophilic bacteria with a prominent immunoevasive capacity, exploiting the immunity for their own growth while shifting the local microbiome into dysbiotic microflora [16, 17]. In context of the healing processes, periopathogens such as *Porphyromonas gingivalis* and *Treponema denticola* interfere with macrophage polarization and cause their shifting from M2 pro-healing to M1 pro-inflammatory course with deleterious effects on the quality of soft tissues and bone implant integration [18–20]. Regarding soft tissues specifically, bacterial colonization of implant surfaces prevents sealing of epithelial cells and down-regulates fibroblast proliferation [7, 14]. From a functional aspect, the lack of periodontal ligament decreases the overall barrier capacity of soft peri-implant tissues and impairs immunological control over local microflora [21, 22], thus facilitating biofilm accumulation, its apical migration, and conversion into more resistant anaerobic population. Due to these reasons, biofilm-free implant surface is considered a key factor to promote successful implant integration.

Implant healing abutments play a pivotal role in implant integration and shaping of supporting soft and hard tissues [14], thus considerable research has been placed on designing and implementing surface treatments with anti-biofilm properties [21–23]. Ghinassi and colleagues noted an improved response of the soft tissues surrounding the laser treated surfaces [24]. Implant surface plasma pre-treatment (PT) is considered promising protocol for dental implants based on their potent anti-biofilm properties, pro-conductive effects on both osteoblasts and fibroblasts and pro-angiogenic effects, collectively providing the optimal conditions for soft and bone integration [23–26].

Previous studies showed that PT removes surface contaminants from titanium abutments more effectively than other available methods, providing a clean surface suitable for cell adhesion [27]. From the physicochemical aspect, this treatment provides surface activation at the atomic and molecular level, enhancing the wettability, cell adhesion, and concomitant spreading to the dental implant surface [28–30]. Moreover, glow discharge PT induces a net-negative charge on the implant surface oxide increasing the functional presentation of the integrin-binding domain, while promoting cell attachment within proportionally accelerated hard and soft tissue integration [31–34]. In the context of dental implants, the unique advantages of PT are its universal applicability to all types of healing abutments, irrespective of their original physicochemical characteristics, and its wide antimicrobial capacity, even against highly virulent bacteria associated with peri-implantitis [32, 35, 36]. In context of soft tissues, a series of in vitro studies showed the capacity of argon plasma to increase migration, proliferation, and adhesion of human fibroblasts to the implant surface, as well as improving effects on soft tissue integration and angiogenesis [37–39]. While an increasing number of in vitro and pre-clinical studies have been published on this treatment, so far very few clinical studies have reported on the outcomes of PT in implantology. Additionally, although the use of high throughput methods is expected to improve the quality of research studies and to accelerate the progress and clinical translation of new biomaterial-based treatments, the use of omics methods is still very rare in periodontology and implantology [40, 41].

Therefore, we hypothesize that pre-treatment of healing abutments by means of argon plasma may positively affect peri-implant microbiome and soft tissue healing processes providing favorable conditions for optimal peri-implant soft tissue integration.

Primary objective of the present study was to estimate the effect of argon plasma pre-treatment (PT) of the healing abutments on peri-implant soft-tissue clinical parameters and microbiome following an initial phase of healing by comparing the clinical and microbiological parameters between treated and non-treated healing abutments after a 3-months healing period. The secondary aim was to investigate the effects of PT of healing abutments with different surface topography (smooth and rough) on soft tissue integration.

## Materials and methods

### Study design

**Fig. 1:**
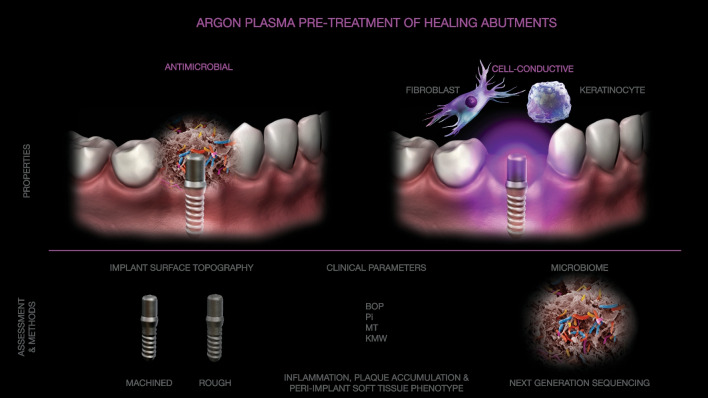
Study design. The study was designed to test hypothesis that argon plasma pre-treatment might provide favorable effects on soft tissue cells and peri-implant microbiome that would contribute to more solid implant soft tissue integration. The standard clinical parameters and peri-implant microbiome were assessed between non-treated and treated healing abutments three months following placement. Additionally, the equal number of abutments with machined and rough surface topography were distributed for each group to secondary assess the effects of experimental treatment of abutments with different surface topography on soft tissue integration within secondary study objective

The present observational study was designed as a proof-of-concept study estimating the effects of argon plasma pre-treatment (PT) of healing abutments on soft tissue integration by comparing the clinical parameters of soft peri-implant tissues and peri-implant microbiome between treated and non-treated implants that were randomly assigned between the groups (Fig. 1). Additionally, to investigate effects of PT on abutments with different surface configuration, the equal number of healing abutments with smooth and rough surface configuration were assigned between the groups according to a second-level randomization schema. The study was conducted in accordance with principles of Helsinki Declaration, experimental protocol was approved by ethics committee (Comitato Etico Lazio 1, Rome, Italy, reference: 813/CE Lazio 1), while patients were informed on study procedures and agreed to participate in the study by signing a written informed consent.

### Study population

The Study population comprised of systemically healthy patients attending the Private clinic in Rome, Italy recruited between January and July 2021. Following periodontal examination, the patients were enrolled in the study if they were systemically healthy and partially edentulous with clear indication for implant placement. Patients were not enrolled in the study if they presented the following exclusion criteria: Implant sites requiring soft or bone tissue grafting; Active periodontal pockets (pocket depth ≥ 4 mm with positive bleeding of probing); Multiple gingival recessions; Intake of antibiotics during follow-up period.

### Healing abutments, antimicrobial surface treatment and randomization

Two types of commercial abutments with different microtopography were used in the study including abutments with smooth surface (MACHINED) and rough ultrathin threaded microsurface (ROUGH) (Sweden & Martina, Padua, Italy). Half of the abutments from each group were randomly selected for treatment using argon plasma in a plasma reactor (Diener Electronic GmbH, Jettingen, Germany) to increase the antimicrobial properties of the surface. Treatment conditions included 75 W of power and − 10 MPa of pressure for 12 min performed immediately before abutment connection as previously described [32]

Two-level randomization schema has been computed for 36 abutments to obtain equal distribution of abutments with smooth surface (MACHINED) and Ultrathin Threaded Microsurface (ROUGH) between experimental and control groups as follows:Argon plasma pre-treated abutments (PT) n = 18: MACHINED = 9, ROUGH = 9; Non plasma treated abutments (NPT) n = 18: MACHINED = 9, ROUGH = 9.

### Clinical assessment of the soft tissue integration quality

The assessment of soft tissue integration was performed based on standard clinical parameters for soft peri-implant tissues including mucosal thickness (MT), keratinized mucosa width (KMW)m and recession depth couples with standard indicators of biofilm accumulation and reactive inflammation including the plaque index (Pi) and bleeding on probing (BOP). Supracrestal mucosal height (SMH) was the single parameter of peri-implant soft tissue phenotype that was not considered, as it would yield erroneous outcomes in stage of healing abutments.

Peri-implant clinical parameters were recorded using a plastic probe graded in millimeters (Colorevue^®^ Probe Williams, Hu-Friedy, Chicago, IL) at six sites per implant by applying 0.15 N/cm force for each representative implant, while the soft tissue phenotype was estimated for each implant as well. All clinical measurements were performed by one experienced examiner (LC) following calibration (96.8% concordance within ± 1 mm for measurements of PD).

**Fig. 2:**
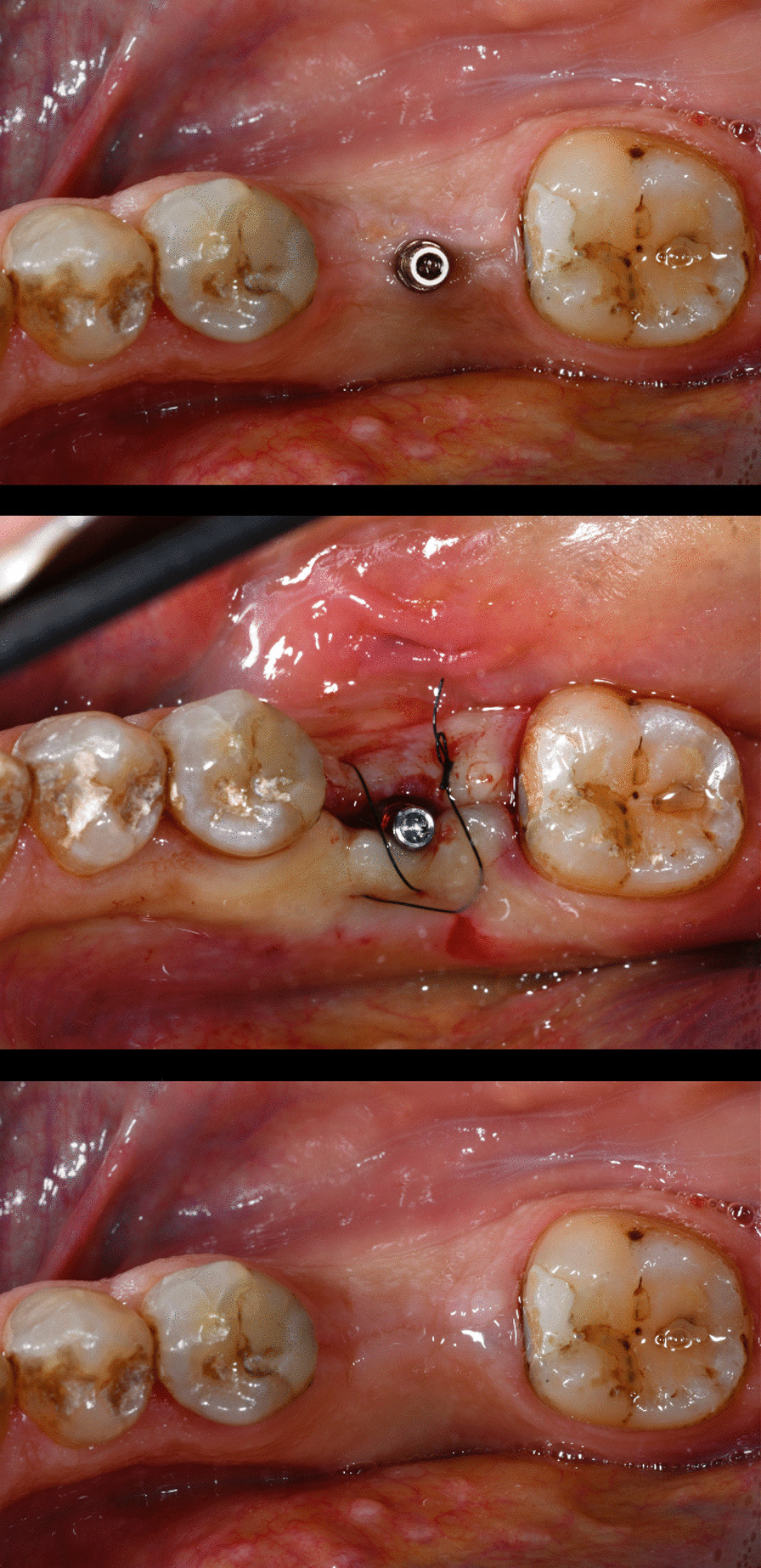
Clinical image before surgery, after the implant insertion and at the healing time

After surgery (Fig. 2), an oral hygiene (OH) home-care regimen was prescribed to the patients following education and patient motivation, which included recommendation for use of adapted interdental hygiene, 7-day use of chlorhexidine (CHX) mouthwash, and usage of an electrical toothbrush.

### Collection of microbiological specimens and assessment of bacterial and fungal peri-implant microbiome

Microbiological samples were collected using GUIDOR^®^ Perio-Implant Diagnostic Test (Sunstar Iberia S.L.U, Barcelona,Spain) with sterile paper points and a 2 ml sterile empty Eppendorf tube according to a previously described protocol [42]. The Bacterial and fungal peri-implant microbiome were assessed using Next Generation DNA Sequencing (NGS). In brief, total genomic DNA was isolated (Institut Clinident SAS, Aix en Provence, France) using the QIAcube^®^ HT Plasticware and QIAamp^®^ 96 DNA QIAcube^®^ HT Kit (Qiagen, Hilden, Germany) according to manufacturer’s recommendations. The elution volume was 150 µl, and twenty-five µl of the extracted DNA was transferred for NGS analysis using the PrecisionBIOME^®^ microbial test (Pangea Laboratory, Tustin, CA, USA). PrecisionBIOME^®^ consists of a targeted NGS workflow including library preparation, sequencing of barcoded amplicons, and bioinformatic analysis of microbial sequences to identify and quantify both bacterial and fungal organisms in a clinical specimen at the species level. Positive and negative controls (buffers only) were also included in the NGS-workflow. ZymoBIOMICS Microbial Community Standard (Zymo Research Corporation, Irvine, CA) was used as a positive control to monitor the performance of all steps of the NGS also including the bioinformatic analysis. Bacterial and fungal amplicons were generated following a targeted approach using the 16S rRNA and ITS2 regions, respectively and the Quick-16S™ NGS library prep kit (Zymo Research Corporation, Irvine, CA) according to manufacturer’s instructions. The NGS library was sequenced using the MiSeq sequencing platform (Illumina, San Diego, CA). The sequencing data was analyzed using a proprietary PrecisionBIOME^®^™ bioinformatics pipeline capable of producing species-level resolution of both bacterial and fungal sequences. Absolute abundance of total bacteria and fungi was determined using the Femto Bacterial and Fungal DNA Quantification kits (Zymo Research Corporation, Irvine, CA) according to manufacturer’s protocols.

### Data management

The primary outcome variables were clinical and microbiological parameters between NPT and PT. Secondary outcome variables were intra-group differences in clinical and microbiological parameters between non treated and treated healing abutments with smooth (MACHINED) and rough (ROUGH) surface topography. Sample size calculation was performed for BOP, thus for a power of 80% the estimated sample size was 25 patients.

Clinical parameters were averaged by site and then calculated by patient and by group. Age and gender were compared using Fisher exact test. The inter-group comparison of clinical parameters was performed using Mann–Whitney test irrespective of data distribution given the relatively small sample size, while the correlation of clinical parameters was performed using Spearman’s correlation test. For the purpose of microbiological clustering, PI and BOP were stratified according to number of positive points as: no = 0; low ≤ 2 and high > 2. α- diversity and evenness were calculated using the Shannon index and the number of observed species. Keratinized gingiva width (KGW) was measured in millimeters and stratified in three clusters as follows: KMW1 < 2 mm, KMW2 = 3–4 mm and KMW3 < 4 mm. β-diversity was calculated using the Bray–Curtis distance at the species taxonomic level. Co-occurrence analyses were used to assess microbial interactions with the standard settings of the R packages “stats v3.6.1″ and “co-occur”, respectively. Analyses of variance and false discovery rate control to correct for type I errors were performed using default parameters of the “vegan” package in R with a *p*-value > 0.05 and the correlation coefficient (r) values of > 0.60 (in absolute values) were considered significant (R version 3.5.2, R Core Team, 2013). Taxonomy heatmaps were generated using a customized Python script to generate hierarchical clustering of samples based on their Bray–Curtis distance. Linear discriminant analysis (LDA) and effect size (LEfSe) were used to identify taxa that were significantly enriched in each group (QIIME version 1.9.1, *p* value > 0.05 was considered significant). The core microbiome was determined based on taxa present with ≥ 0.1% relative abundance in ≥ 50% of all samples. Species that had a relative abundance of at least 1% in each data set were used in this analysis, and data was visualized using the same software used for statistical analyses (Prism 8.0, GraphPad Software, La Jolla, CA).

## Results

The final sample size consisted of 30 periodontally stable patients that completed the study follow-up since four patients (one in each group) had to be excluded because of antibiotic intake during the follow-up period, while two patients (PT-MACHINED) were excluded based on NGS quality control criteria (DNA amplification cycle threshold value greater than negative control > 35). Due to limited sample size, the microbiome analysis is provided only for primary outcome variables, while the microbiome outcomes for the healing abutments with different topographies were not considered for interpretation but they are provided as Additional file 1. Demographic and clinical parameters by groups are shown in Table 1.

**Table 1:** Clinical parameters between the groups

	NPT	PT	Machined	Machined + PT	Rough	Rough + PT
*Gender*						
Male (%)	50	64.3	62.5	83.3	37.5	50
Female (%)	50	35.7	37.5	16.7	62.5	50
Age	55.38 ± 14.71	50.04 ± 10.23	61.88 ± 16.2	42.2 ± 15.8	48.88 ± 12.7	57.88 ± 10.5
MT	NS		Machined > Machined + PT; p < 0.037		NS	
Thin	18.75 ± 6.12	57.14 ± 0.9	75 ± 6.78	50 ± 6,7	75 ± 6,7	50 ± 6,7
Thick	81.25 ± 18.93	42.85 ± 3.2	25 ± 6.49	49 ± 1,2	25 ± 6,4	49 ± 7,1
KGW (mm)	NS		NS		NS	
	3.40 ± 0.910	3.58 ± 0.996	3.57 ± .1.134	3.71 ± 0.983	3.44 ± 882	2.86 ± 0.707
BOP	NPT > PT; *p* = 0.036		NS		Rough > Rough + PT; *p* = 0.000	
	13.2 ± 35.2	0.00 ± 0.00	0.00 ± 0.00	0.00 ± 0.00	25.1 ± 46.3	0.00 ± 0.00
Pi	NPT > PT; *p* = 0.021		Machined > PT/Machined *p* = 0.004		NS	
	40.1 ± 15.07	20.8 ± 14.3	43.2 ± 23.5	14.1 ± .17.8	38.4 ± 15.18	25.1 ± .18.9

Values are expressed as % mean ± SD except for gender distribution

*PT*, plasma pre-treatment; *NPT*, no plasma pre-treatment; *MT*, mucosal thickness; *KMW*, keratinized mucosa width; *BOP*, bleeding on probing; *Pi*, plaque index, *NS*, not significant

### Clinical parameters between different abutment surfaces

The comparison of clinical parameters between the groups is listed in Table 1. NPT group showed increased BOP (*p* = 0.036) and PI (*p* = 0.021) values while the MT and KMW were not affected by the treatment. Regarding the effects of PT on abutments with different configuration, NPT-MACHINED showed higher PI (*p* = 0.004) and more frequent thick tissues (*p* = 0.037) compared to PT-MACHINED, while NPT-ROUGH exhibited significantly higher BOP (*p* < 0.000) when compared to PT-ROUGH. Significant positive correlation was established between BOP and Pi (*p* = 0.025), while the BOP was negatively correlated with KMW (*p* = 0.045).

### Peri-implant microbiome between NPT and PT healing abutments

**Fig. 3:**
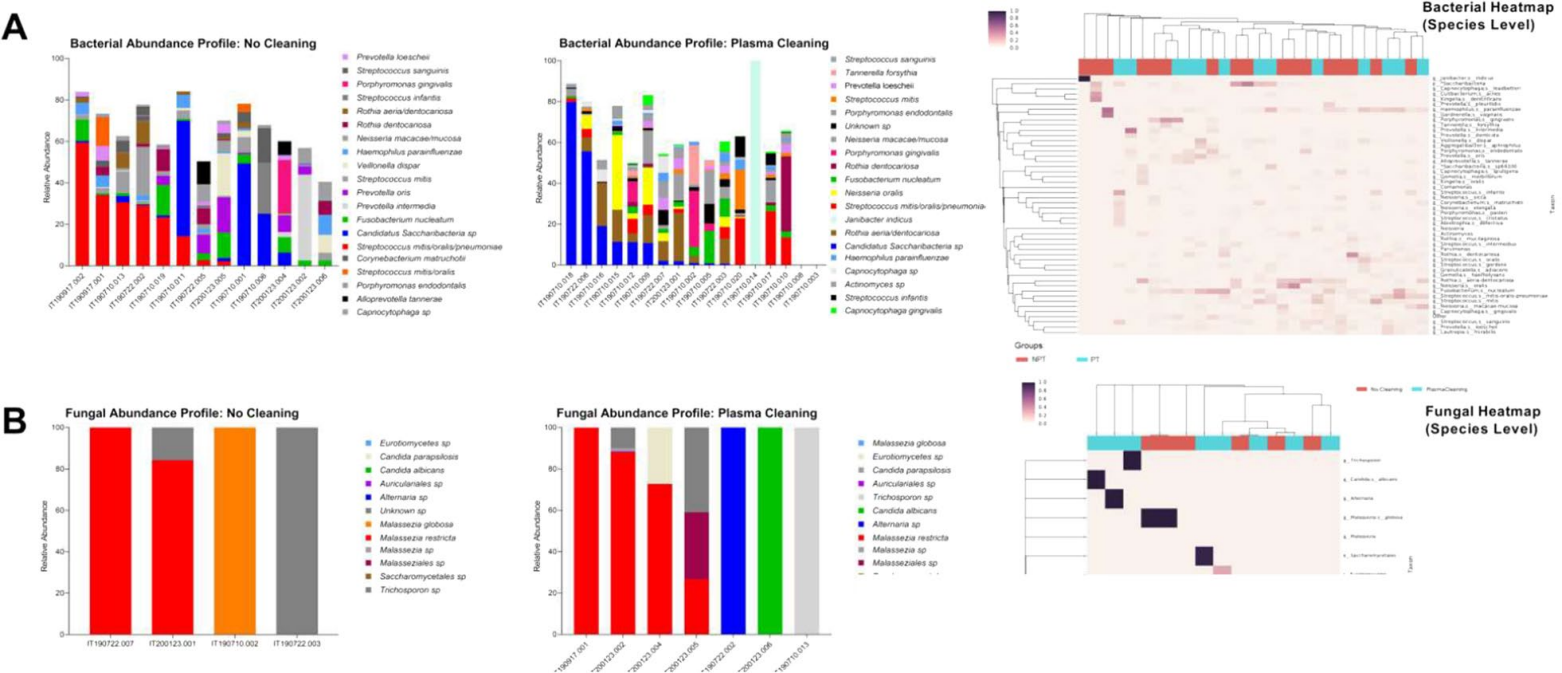
Peri-implant microbiome between the groups including bacterial (**A**) and fungal (**B**) species abundance with *respective* taxonomy heatmaps showing the bacterial and fungal diversity at the species level for the four-group comparison following applied sample clustering. A total of 283 different bacterial and 11 fungal species were detected in the datasets

**Fig. 4:**
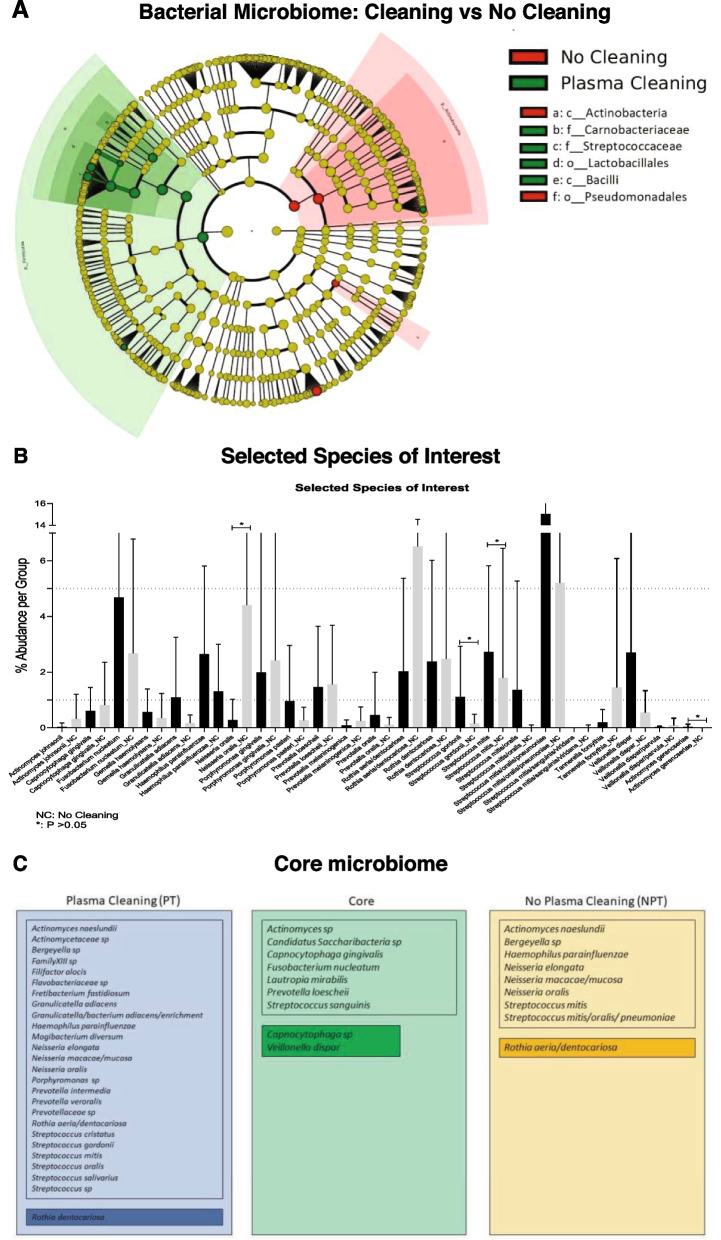
Differences in microbial profiles between PT and NPT groups and core microbiome. **A** Cladogram generated by LEfSe analysis highlights differences in taxa between argon plasma pre-treated (PT) and non-pre-treated (NPT) healing abutments (*p* > 0.05). Regions in red indicate taxa that are significantly enriched in the NPT, while regions in green represent taxa enriched in PT. Nods colored in yellow are not significantly different between groups. Each ring represents a taxonomic level, with the phylum level on the inside and the genus level in the outermost ring. **B** Differences in abundance amongst bacterial species of interest detected in PT versus NPT groups are expressed as bar plots indicating the average relative abundance and standard deviation for each species in the PT (grey) and the NPT group (back). In general, PT increased abundance of *Streptococcus gordonii*, *Streptococcus mitis*, and *Actinomyces gerencseriae*, while NPT healing abutments exhibited increased abundance of *Neisseria oralis.* The fungal diversity was higher in PT when compared to NPT, but without statistical significance. Marked with an asterisk are the species significantly different between groups (*p* < 0.05). **C** Core microbiome analysis panels show the species that are (1) part of a core, shared microbiome between PT and NPT groups (in green), (2) unique for PT group (in blue), and (3) unique for NPT group (in yellow). The core microbiome was calculated as described in the methods section. Briefly, the species had to be present in more than 50% of patients in each group, and at an average abundance of at least 0.1%. Highlighted in a darker shade are those species that were present in at least 2% of relative abundance or higher

The alpha and beta diversities between NPT and PT a were not significantly different between the groups for either bacterial or fungal microbiome (*p* > 0.05). Peri-implant microbiome analysis showed a diverse bacterial microbiome consisted of 283 different species, and less diverse mycobiome comprising 11 different fungal species (Fig. 3). While bacteria were detected in all samples, fungal species were detected in only 47% of the specimens (14/30). The bacterial (Fig. 3A) and fungal (Fig. 3B) microbiome was characterized by a large degree of inter-sample diversity. Plasma cleaning significantly influenced peri-implant bacterial microbiome at different taxonomic levels (Fig. 4A), while the fungal microbiome remained unaltered and is thus excluded from depiction. At the phylum level, *Firmicutes* were significantly more abundant in the PT group (*p* = 0.014), while *Actinobacteria* were significantly more abundant in the NPT group (*p* = 0.038). *Saccharibacteria* were more prevalent in the NPT group, but this difference was not statistically significant. At the species level, the most abundant bacterial species detected in the PT group were *Streptococcus mitis/oralis/pneumoniae*, *Candidatus saccharibacteria* sp., and *Fusobacterium nucleatum*. In the NPT group, the most abundant species were *Candidatus saccharibacteria* sp., *Rothia aeria/dentocariosa,* and *Janibacter indicus*. Four bacterial species were significantly different between the groups including *Actinomyces gerencseriae*, *Streptococcus gordonii,* and *Streptococcus mitis* that were increased in PT, while *Neisseria oralis* was more abundant in NPT (Fig. 4B; *p* < 0.05).

Regarding the cluster of periodontal pathogens and commensals, the frequencies and their relative abundances were not significantly different between the groups, but according to LefSe analysis, *Granulicatella adiacens, Rothia dentocariosa,* and *Haemophilus parainfluenzae* were more prevalent in the PT group, while *Rothia aeria/dentocariosa* and *Streptococcus mitis/oralis/pneumoniae* were more prevalent in the NPT group. The shared core microbiome between the groups have been identified and consisted of *Capnocytophaga* gingivalis, *Fusobacterium nucleatum*, *Lautropia mirabilis*, *Prevotella loescheii,* and *Veillonella dispar* (Fig. 4C).

The fungal diversity was higher in PT when compared to NPT, but without statistical significance. The most common fungal species in both groups was *Malassezia restricta*.

**Fig. 5:**
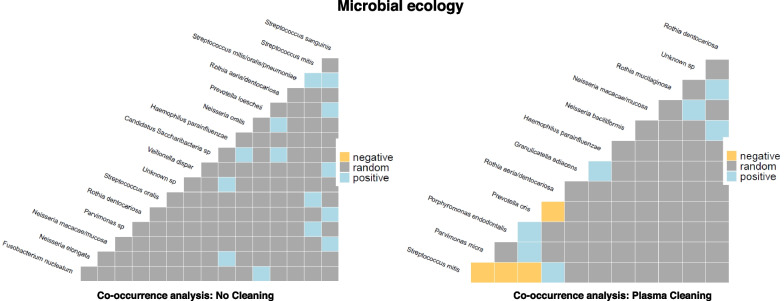
Microbial ecology and bacterial co-occurrence. The most expressed interactions were observed between *Streptococcus* species. In the NPT were observed only positive interactions for *Streptococcus mitis/oralis/pneumoniae, Rothia aeria/dentocariosa, Prevotella loescheii, Haemophilus parainfluenzae, Veillonella dispar, Rothia dentocariosa, Fusobacterium nucleatum, Streptococcus mitis* and *Neisseria oralis.* In the PT group, *Streptococcus mitis* was positively associated with *Rothia aeria/dentocariosa* and negatively associated with *Parvimonas micra*, *Porphyromonas endodontalis* and *Prevotella oris*. Negative interactions between species are depicted in yellow, positive interactions in blue and insignificant interaction in grey

While there were no statistically significant interactions between fungal and bacterial species, significant co-occurrence was observed between bacterial species (Fig. 5). Generally, the most prominent interactions were observed between *Streptococcus* species suggesting a symbiotic relationship between these species. In the NPT, was observed only positive interactions for *Streptococcus mitis/oralis/pneumoniae, Rothia aeria/dentocariosa, Prevotella loescheii, Haemophilus parainfluenzae, Veillonella dispar, Rothia dentocariosa, Fusobacterium nucleatum, Streptococcus mitis* and *Neisseria oralis.* In the PT group, *Streptococcus mitis* was positively associated with *Rothia aeria/dentocariosa* and negatively associated with *Parvimonas micra*, *Porphyromonas endodontalis,* and *Prevotella oris*.

### Association between clinical parameters and peri-implant microbiome

**Fig. 6:**
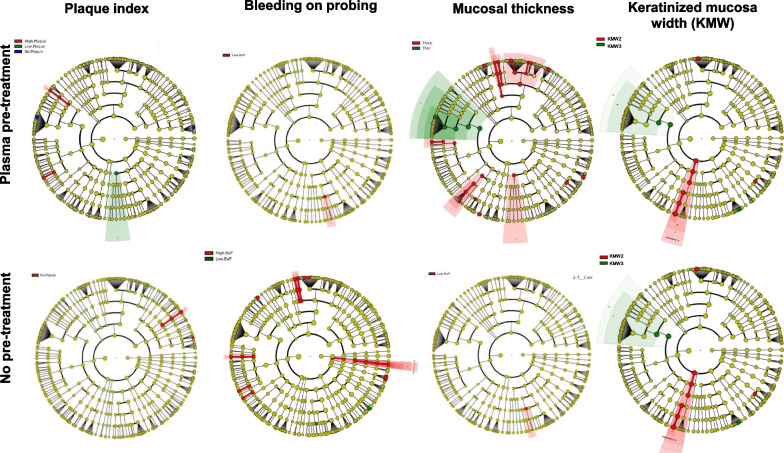
LEfSe analysis associating bacterial species with clinical parameters. Cladograms depict taxa that were significantly different between the clinical parameters at different taxonomic levels (*p* > 0.05). Each ring represents a taxonomic level, with the phylum level on the inside and the genus level in the outermost ring. Nods colored in yellow are not significantly different between groups. In the PT group *Stomatobaculum longum* and *Staphylococcus epidermidis*, were associated with high plaque accumulation (*p* = 0.027), while the *Streptococcus intermedius* (*p* = 0.033) and *Actinomyces naeslundii* (*p* = 0.049) were associated with low plaque accumulation. Thin phenotype was positively correlated with *S. gordonii* in PT (*p* = 0.030). In NPT group lack of plaque was associated with *Janibacter indicus* and *Schaalia* sp. (*p* = 0.0236), the high BOP was associated with *Treponema denticola* (*p* = 0.035), *Tannerella forsythia* (*p* = 0.021) and *Prevotella oralis* (*p* = 0.040), while there were no statistically significant associations between microorganisms and phenotype. As only one patient per group exhibited KMW 1 values the association of microbiome with KMW was assessed only for KMW 2 (3–4 mm) and KMW 3 (> 4 mm). In NPT 22 different genera and 46 different species were significantly more frequent in KMW 3 compared to KMW 2, while 9/46 species were of the genus *Streptococcus*. In PT three species were significantly different between the two groups, *Neisseria sicca* was significantly enriched in KMW 3 (*p* = 0.011); while *Prevotella oris* (*p* = 0.024) and *Fusobacterium nucleatum* (*p* = 0.033), more abundant in KMW 2. No fungal taxa were significantly different between the groups

Bacteria identified as discriminants between PT and NPT according to LefSe analysis were correlated with clinical parameters (Fig. 6). In the PT group, *Stomatobaculum longum* and *Staphylococcus epidermidis*, were associated with high plaque accumulation (*p* = 0.027), while the *Streptococcus intermedius* (*p* = 0.033) and *Actinomyces naeslundii* (*p* = 0.049) were associated with low plaque accumulation. While there was no specific correlation between BOP and microbial signatures, the thin phenotype was positively correlated with *S. gordonii* in PT (*p* = 0.030). In the NPT group, lack of plaque was associated with *Janibacter indicus* and *Schaalia* sp. (*p* = 0.023, the high BOP was associated with *Treponema denticola* (*p* = 0.035), *Tannerella forsythia* (*p* = 0.021) and *Prevotella oralis* (*p* = 0.040), while there were no statistically significant associations between microorganisms and phenotypes. The association of microbiome with KMW was assessed only for KMW 2 and KMW 3 since only one patient in NPT and in the PT group exhibited KMW < 2 mm (KMW1). In NPT 22 different genera and 46 different species were significantly more frequent in KMW 3 compared to KMW 2, while 9/46 species were of the genus *Streptococcus*. In PT the LEfse analysis showed that three species were significantly different between the groups, *Neisseria sicca* was significantly enriched in KMW 3 (*p* = 0.011); while *Prevotella oris* and *Fusobacterium nucleatum* (*p* = 0.024 and *p* = 0.033, respectively), more abundant in KMW 2. No fungal taxa were significantly different between the groups.

## Discussion

Present study demonstrated that argon plasma pre-treatment of healing abutments contributes to the lower soft tissue inflammation and plaque accumulation, Further, it prevents advanced biofilm formation by favorizing predominance of pioneer colonizers over late colonizers that were in turn significantly more abundant around non-treated abutments. PT did not affect soft tissue phenotype at the clinical level, but at the microbiome level was established the association between *S. gordonii* and thin tissue in PT, and regarding KMW, the abundance of periopathogenic bacteria including *Prevotella oris* and *Fusobacterium nucleatum* were observed in patients with narrower KMW in PT.

The advances in biofilm research driven by breakthrough in molecular techniques have changed the face of oral microbiology, revealing far higher diversity of peri-implant microflora with complex interactions and functional behavior in both health and disease [43, 44]. In the context of antimicrobial materials, it is considered that microbiome research may pave the way for optimal anti-infective treatments by unveiling key treatment targets while allowing highly accurate estimation of treatment effectiveness [40, 45]. Finally, it is considered that omics methods are gamechangers in tackling a double-sided complexity of biological systems and material properties, able to provide the highly accurate and sensitive assessment of material-induced biological effects, accelerating its clinical translation for therapeutic use [41, 46]. Hence, the clinical metagenomics becomes indispensable tool for assessment of infective pathologies and their responsiveness on performed treatments in dentistry [47]. The present study has demonstrated a highly diverse microbiome consisting of 283 different bacterial species around healthy implants following a period of initial integration, as previously reported [11, 40, 41]. Within these species, both early and late colonizers, as well as key pathogens were already present two months post-implantation. This finding is in discordance with previous reports that showed presence of key pathogens only six months following implantation [20]. These findings once again confirm the importance of rigorous infection control from the initial healing stage following implant placement [7]. The fungal microbiome is currently in the spotlight of biofilm research related to prominent capacity of fungal species to form polymicrobial biofilms with a diverse spectrum of bacterial species (particularly with streptococcal species) thus contributing to the accumulation and maturation of dysbiotic biofilms [48, 49]. The mycobiome was generally less diverse than bacterial community and *Malassezia restricta* represented the most prevalent fungi species as previously reported [50, 51], although its exact role in the oral ecosystem is still unknown.

PT improved the clinical parameters of soft tissue inflammation and plaque accumulation, and positively affected peri-implant microbiome, preventing the growth of late colonizers and related biofilm maturation. PT exhibited higher abundance of pioneer or early colonizers such as *S. gordonii* and *S.mitis* [46, 47] as well as *Actinomyces* known to successfully congregate with *S. gordonii* [52]. In contrast, the NPT group showed a higher abundance of late colonizers like *Neisseria oralis* [53] suggesting an advanced stage of biofilm formation in NPT. The higher abundance of bacterial species on plasma treated surfaces might be explained by the fact that this treatment increases the surface wettability, which increases cell-attractiveness, even though this treatment was clearly associated with lower plaque accumulation and less inflammation. Future studies are however needed to decipher the specific role of *S. gordonii*, which is considered a member of the healthy microflora, but also forms synergistic biofilms with *Parvimonas micra* and *Fusobacterium nucleatum,* which in turn are associated with peri-implantitis [42, 54]. Given the fact that *Fusobacterium nucleatum* was also identified as a member of the core microbiome, shows the importance of timely biofilm control. The high-dimensional class comparison using LEfSE method is praised for its capacity to determine the microbiological characteristics/associations relevant for understanding the inter-group differences and the biological relevance/impact on oral health [55]. In this study we found a significant co-occurrence between *S.mitis* and *Rothia dentocariosa* and co-exclusion between *S.mitis* and pathogenic species including *P. micra*, *Porphyromonas endodontalis,* and *Prevotella oris*. This confirms the pattern of peri-implant health associated with Gram-positive cocci and pathological conversion that follows the competitive shift caused by anaerobic species [56, 57]. On the other hand, the NPT group showed a significant increase in plaque accumulation and higher inflammation index values, which were associated with *Tanerella forsythia* and *P. oris* comparable to peri-implant mucositis [58, 59]. These results point to the beneficial effects of PT in preventing plaque-induced peri-implant inflammation. However, the more prevalent thin tissue and its established association with abundance of *S. gordonii* in the PT group requires further investigation.

Although a number of in vitro studies demonstrated the capacity of argon plasma to increase migration, proliferation and adhesion of fibroblasts to the titanium surface [37, 39], the PT did not show specific effect on KGW and MT suggesting rather indirect effect of this pre-treatment in providing favorable conditions for soft-tissue integration than direct pro-conductive effect. However, the variable microbial signatures were associated to different size of KGW. It would be thus worth to disclose possible interfering effects of this periopathogen with soft tissue collagen synthesis since it has been recently suggested that fibroblast plasticity around titanium implants might be altered by the surface characteristics and by the nature of the inflammatory stimuli [60, 61].

Within commitment of the modern health care toward focused cutting-edge therapies for an increased therapeutic index, tackling the complex biologics and its measurement remains the major prerequisite. It is now established that available regenerative procedures in periodontology and implantology provides in average up to 50% of complete success despite autologous origin or high-grade biomimetic concept, mostly due to altered healing processes and the presence of local interfering factors (such as microbial). It is thus proposed that bioactive approaches targeting specific biological factors should favorize healing processes and yield improved treatment outcomes [62, 67]. On the other hand, the rise of precision medicine has been driven by innovations in molecular profiling specifically for biomaterials, as high-throughput methods allowed tackling the complexity of interaction between biomaterials and biologics and allows for a better understanding of correlations between material properties and their effects on complex biological systems. Thus the personalized strategies and use of omics methods from discovery, over development, preclinical evaluation, and implementation of new clinical approaches are holding the promise to overcome erroneous material design and validation methods usually confined to the low-through put approach behind therapies with limited capacity and represents subject of strongest recommendation in medicine and dentistry [68, 69].

Biomaterial-related infections are considered as the most devastating complication of biomaterial use associated with ravaging biological consequences, hence development of anti-infective biomaterials and infection-resistant surfaces is one of the major priorities in biomedical research [70]. The present study has demonstrated that peri-implant microbiome remains highly diverse already during the first month post-implantation in clinically healthy peri-implant tissues, emphasizing the importance of biofilm control from the early stage of implant placement. The anti-infective pre-treatment of healing abutments seems to be a promising approach providing favorable conditions for optimal soft tissue growth and seal able to withstand infective threats. Furthermore, the controversial findings of lower inflammation coupled thin mucosal tissue and high prevalence of *Streptococcus gordonii* observed in PT confirms the complexity of material-induced biological effects and the critical role of high throughput methods for accurate evaluation of treatment effectiveness and its rapid clinical translation [41, 46]. Thus, the accelerated implementation of comprehensive biological assessment in dental research undoubtfully remains a roadmap toward improved treatment strategies in implantology [44, 47]. Although this study did not primarily aim to assess the effect of PT on abutments with different topography, the findings of decreased BOP in ROUGH + PT compared to ROUGH healing abutments is suggestive that this surface treatment may attenuated reported upregulated inflammatory response, reported for rough surface topography (1). Additionally, the lower Pi was confirmed on MACHINED + PT compared MACHINED, hence considering observed differences between abutments, this proof-of-concept study provides justification for conducting the further research primary oriented toward effects on rough and machined surface abutments in larger sample. Moreover, given the similarity in opportunistic pathobionts identified between peri-implant microbiome and peri-prosthetic infections in orthopedics (such as *Staphylococcus aureus*) [54, 71], the findings in this study may be transferable toother medical fields, representing another implication for future research.

The present study exhibits some limitations, primary relating to the relatively small sample size and lack assessment of collagen markers that would possibly provide more specific information regarding treatment effects on soft tissue metabolism. Finally, the assessment of biological parameters should represent an integral part of evaluation protocols in pre-clinical and clinical studies assessing soft peri-implant tissues as being able to provide more precise information over the sensitivity of standard clinical parameters.

## Conclusion

Within limitations of this study, argon plasma pre-treatment of healing abutments provided positive effects on peri-implant microbiome resulting in decreased biofilm accumulation and lower soft-tissue inflammation, while the qualitative PiSP seems to be unaltered by this protocol.

## Supplementary Information

**Additional file 1**: (1) Alpha and beta diversities to assess the microbiome composition. (2) Linear discriminant analysis (LDA) effect size (LEfSe) analysis of bacterial composition changes due to treatment of the patient. (3) Heatmaps.

## Data Availability

The raw/processed data required to reproduce these findings cannot be shared at this time as the data also forms part of an ongoing study.
