# Supplementary material for: Effect of argon plasma pre-treatment of healing abutments on peri-implant microbiome and soft tissue integration: a proof-of-concept randomized study
Source: BMC Oral Health. 2023 Jan 17;23:27. doi: 10.1186/s12903-023-02729-1 (PMC9843976; doi:10.1186/s12903-023-02729-1)
Supplement: Supplementary file 1 — Additional file 1: (1) Alpha and beta diversities to assess the microbiome composition. (2) Linear discriminant analysis (LDA) effect size (LEfSe) analysis of bacterial composition changes due to treatment of the patient. (3) Heatmaps. [file 12903_2023_2729_MOESM1_ESM.docx]

**Supplementary material**

**The effect of argon plasma pre-treatment on peri-implant microbiome around healing abutments with different surface topography.** MAC-machined; Plasma MAC-plasma pre-treated machined; UTM-rough; Plasma UTM-plasma pre-treated rough. No statistical difference was observed for any comparison p>0.05.

Alpha and beta diversities are commonly used markers to assess the microbiome composition of a biological habitat. The alpha diversity measures the diversity within a sample, while beta diversity measures the diversity between samples.

The graph on the left shows the alpha diversity of each group as calculated by the number of observed bacterial species per sample; the graph on the right the beta diversity as calculated by Bray–Curtis-distance at the species for the bacterial composition of all samples. Color coding is consistent for both groups to indicate the treatment groups. There was no statistically significant difference in the alpha or beta diversities between groups (p>0.05).

Shown are the results of the linear discriminant analysis (LDA) effect size (LEfSe) analysis of bacterial composition changes due to treatment of the patient. The cladograms

indicates taxa that are significantly enriched in the healthy group, regions in red those significantly enriched in MAC, regions in green represent taxa enriched in Plasma MAC,

regions in blue those for the UTM group, and regions in purple taxa that are significantly enriched in the Plasma UTM group. Nods colored in yellow are not significantly different between groups. Each ring represents a taxonomic level, with the phylum level on the inside and the genus level in the outermost ring. Taxa with significantly higher abundances are listed on the right side of the graph, with only five taxa being enriched in the UTM group.

Heatmaps of the microbial composition for bacteria (top) and fungi (bottom). The top 50 species of highest relative abundances are shown in rows. Samples shown in columns were clustered hierarchically based on Bray-Curtis distances (PCoA).

Heatmaps
